# Lessons for the clinical nephrologist: recurrent pregnancy-associated thrombotic microangiopathy (TMA) with a known complement mutation and features of preeclampsia; a diagnostic and therapeutic dilemma

**DOI:** 10.1007/s40620-021-01035-9

**Published:** 2021-06-07

**Authors:** Priscilla Smith, Alyaa Abdelmaguid, Katherine Clark, Kate Bramham

**Affiliations:** 1 King’s Kidney Care, King’s College Hospital and King’s College London, NHS Foundation Trust, Denmark Hill, London, SE5 9RS UK; 2 Medical Research Institute, Alexandria University, Alexandria, Egypt; 3 Department of Women and Children’s Health, King’s College London, London, UK

**Keywords:** Pregnancy, Thrombotic microangiopathy, TMA, aHUS, Preeclampsia

## The case

**Fig. 1:**
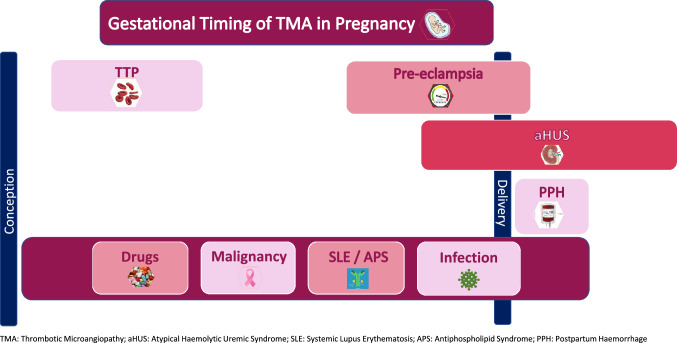
Gestational timing of thrombotic microangiopathy in pregnancy. *TMA* thrombotic microangiopathy, *aHUS* atypical haemolytic uremic syndrome, *SLE* systemic lupus erythematosus, *APS* antiphospholipid syndrome, *PPH* postpartum haemorrhage

We report a case of recurrent thrombotic microangiopathy (TMA) in pregnancy in a 22-year-old with previous liver transplantation for congenital hepatic fibrosis. She was taking insulin and labetalol for chronic hypertension and gestational diabetes, and tacrolimus. She presented at 18-weeks’ gestation with generalized swelling, breathlessness, 30 kg weight gain, hypertension (150/100 mmHg), proteinuria (urinary protein: creatinine ratio (uPCR) 201.3 mg/mmol) with acute kidney injury, deranged liver function tests, thrombocytopenia and biochemical and haematological features of TMA (Table 1). Chest X-ray showed left pleural effusion, but renal ultrasound, lower limb Dopplers and echocardiogram were normal. Differential diagnoses for pregnancy-associated TMA were considered (Fig. 1).

**Table 1:** Results (pregnancy bloods are the most extreme results recorded)

	2015 Non-pregnant	2016 Pregnancy	2018 Pregnancy	2019 Non-Pregnant
Serum creatinine (µmol/l)	52	126 ↑	199 ↑	69
Haemoglobin (g/dl)	126	94 ↓	87 ↓	138
Platelets (cells/10^9^ µl)	72	13 ↓	43 ↓	89
Haptoglobin (mg/dl)	–	< 0.1 ↓	< 0.1 ↓	–
Blood film	–	Occasional fragments	No significant fragments	–
Absolute reticulocyte count (10^9^/l)	–	311.5 ↑	482.6 ↑	–
Lactate dehydrogenase (U/l)	–	557 ↑	559 ↑	–
Direct antiglobulin test	–	Negative	Negative	–
Total bilirubin (µmol/l)	27	65 ↑	57 ↑	37
Aspartate transaminase (U/l)	51	79 ↑	101 ↑	38
Activated partial thromboplastin time ratio	1.11	1.2 ↑	1.18 ↑	1.07
Tacrolimus level (μg/l)	5	12.6 ↑	10.9 ↑	8.0
C3 (g/l)	0.58	0.61 ↓	0.61 ↓	0.66
C4 (g/l)	0.14	0.10 ↓	0.07 ↓	–
Anti-dsDNA IU/ml	–	1	< 1	–

Rise in creatinine occurred prior to tacrolimus level increase

Thrombotic Thrombocytopenic Purpura was excluded (ADAMTS-13 activity 22%), connective tissue, autoimmune and thrombophilia investigations were negative (Antiphospholipid-antibodies, ANA, Anti-dsDNA, anti-Scl-70, Anti-GBM (< 0.8 U/ml), C3-nephritic factor, prothrombin gene and factor-V Leiden gene). Tacrolimus concentration was within the therapeutic target range (8.8 µg/l). Renal biopsy was not feasible due to clinical status and low platelet count. Intrauterine death occurred at 22-weeks’ gestation. Subsequent genetic testing confirmed complement mutation (heterozygous c.1855G > A(p.Val619Met) variant in exon 15; C3 gene), which was not present in stored liver donor DNA.

Pre-pregnancy counselling was provided by a specialist nephrologist and obstetrician about future pregnancy risk. Approval for eculizumab use in future pregnancies if TMA reoccurred was obtained in view of the complement mutation and potential risk of recurrent disease [1] but prophylaxis was not advised. She was vaccinated against pneumococcal and meningococcal infections.

In her next pregnancy (18 months later), she developed recurrent TMA at 22 weeks’ gestation [serum creatinine 199 µmol/l (baseline 60 µmol/l), platelets 50 × 10^9^/l and anaemia (Hb 107 g/l)]. Blood pressure was 160/100 mmHg, and she had substantial peripheral oedema. Other investigations were unremarkable and fetal scans were reassuring. Placental Growth Factor concentration (PLGF) was low < 12 pg/ml.

**Fig. 2:**
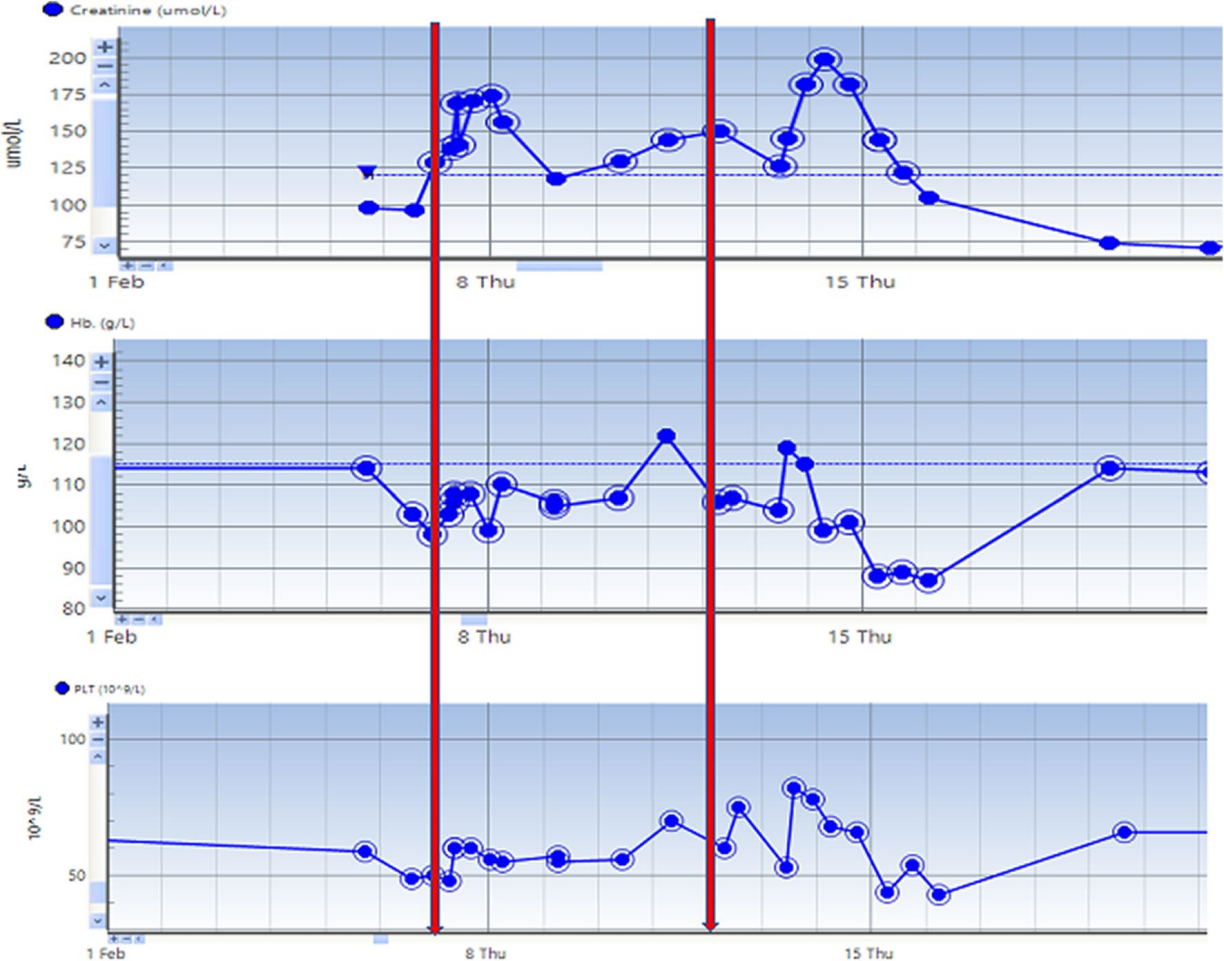
Serum creatinine, haemoglobin and platelet changes in second pregnancy

Eculizumab (900 mg) was commenced. However, renal function continued to deteriorate, so a second dose (1200 mg) was given on day 5 which led to stabilization and improvement in all markers (Fig. 2). Transient creatinine rise following dose was attributed to concurrent infection. Fetal demise occurred on day 8 of treatment. Placental histology showed “features of hypoperfusion including intervillous fibrin deposition, fibrin thrombi and foci of ischaemic type necrosis. Calcification and syncytial knots noted, which is suggestive of pre-eclampsia”.

Uncertainty surrounding the diagnosis of preeclampsia or aHUS was communicated and counselling regarding potential risks and benefits of prophylactic eculizumab in a future pregnancy was provided. She was advised to use effective contraception and is considering surrogacy for future pregnancies.

## Discussion

### What is the association between pregnancy and aHUS?

Atypical Haemolytic Uraemic Syndrome (aHUS) is rare and estimated to affect 1 in 25,000 pregnancies, typically presenting with acute kidney injury (AKI), microangiopathic haemolytic anaemia and thrombocytopenia [1], and is associated with substantial morbidity and mortality [2]. Primary aHUS is associated with complement gene mutations [3], and 21% of cases in women of childbearing age are associated with pregnancy [4]. Diagnosis is challenging due to common features with pre-eclampsia, connective tissue disorders and thrombotic thrombocytopenic purpura. Early confirmation of TMA aetiology is critical to facilitate appropriate management.

### Could this be a drug-induced TMA?

Tacrolimus may cause TMA, but reported cases are few and pathophysiology remains uncertain. Idiosyncratic immune reactions and dose-related phenomena are reported even with therapeutic concentrations [S1]. Endothelial activation and platelet aggregation associated with tacrolimus may lead to TMA, which usually resolve with withdrawal; however, TMA may be refractory to withdrawal associated with complement activation. In this case, the tacrolimus dose was stable and trough levels were therapeutic (8.8 µg/l), which does not preclude a drug effect, but TMA resolution postpartum despite continuing tacrolimus makes this trigger less likely.

### How do liver transplantation and pregnancy affect complement production?

In silico analysis of the reported complement mutation suggested the variant may be a rare benign polymorphism [5], but deleterious effects were not excluded. The liver produces > 90% of circulating C3 [6], but transplant recipients with discordant C3-allotypes have extrahepatic C3 upregulation for up to a year post-transplant [7]. Liver transplantation occurred 4-years prior, but pregnancy-associated upregulation of extrahepatic complement may increase the impact of the recipient C3 mutation.

### Can you use Eculizumab in pregnancy?

Eculizumab is not licensed for use in pregnancy, but several cases have now been reported with successful renal outcomes [8]. However, fetal and neonatal mortality associated with pregnancy-associated TMA is high and we propose that disease processes rather than eculizumab therapy led to fetal demise. In view of assumed pregnancy-associated plasma volume expansion, a higher 2nd induction dose (1200 mg) was used, with haematological and biochemical response. In keeping with our case, others with pregnancy-associated TMA have successfully stopped treatment without recurrence [9], but optimal timing and risk of relapse remains unknown.

Eculizumab has been detected in umbilical cord blood [S2], no adverse short-term effects have been reported but long-term outcomes are undetermined [S3].

### Is this all just preeclampsia?

Diagnostic uncertainty of pregnancy-associated TMA is highlighted. Low PlGF and placental histology were suggestive of placental insufficiency; however, placental impact of the complement mutation is unclear. Prolongation of pregnancy with renal recovery in severe early preeclampsia has been reported in postpartum TMA with eculizumab treatment [S4–6]. A phase-1 trial of eculizumab in Haemolysis-Elevated-Liver-enzymes-Low-Platelet Syndrome may provide further insight [S7]. Better data describing temporal changes in TMA parameters in pregnancy are likely to facilitate earlier diagnosis and improve maternal and neonatal outcomes.
